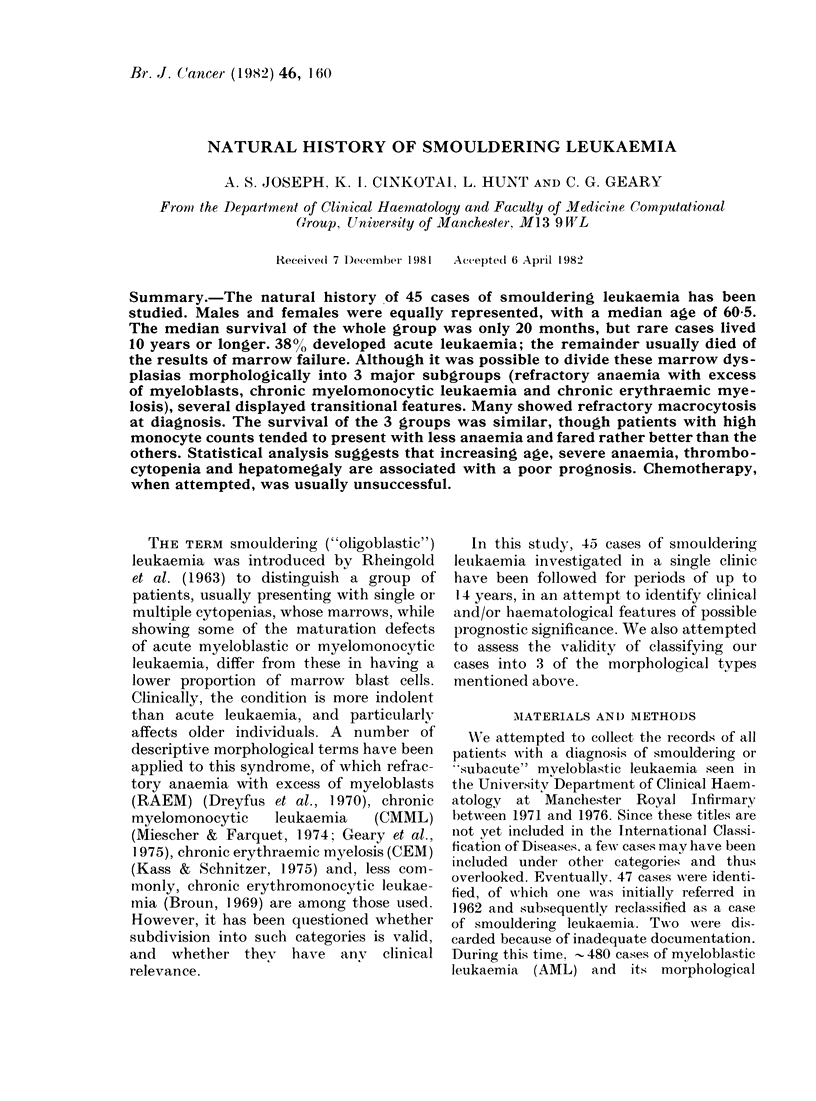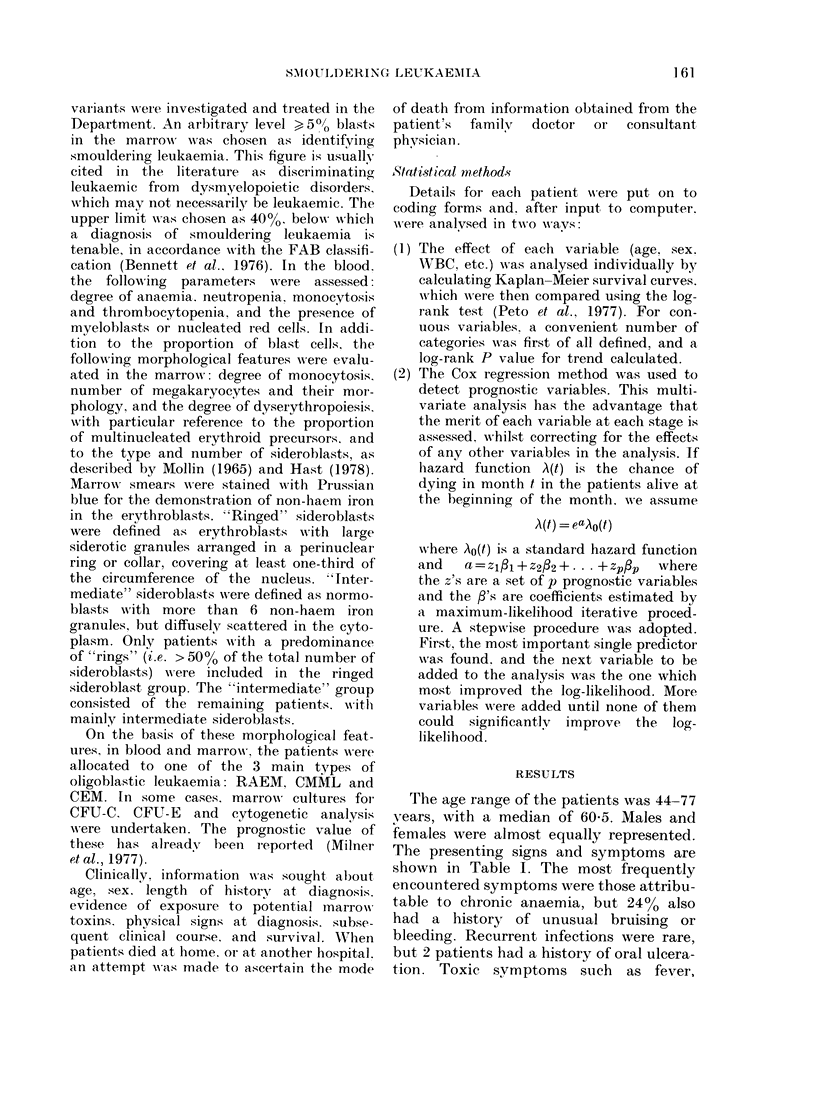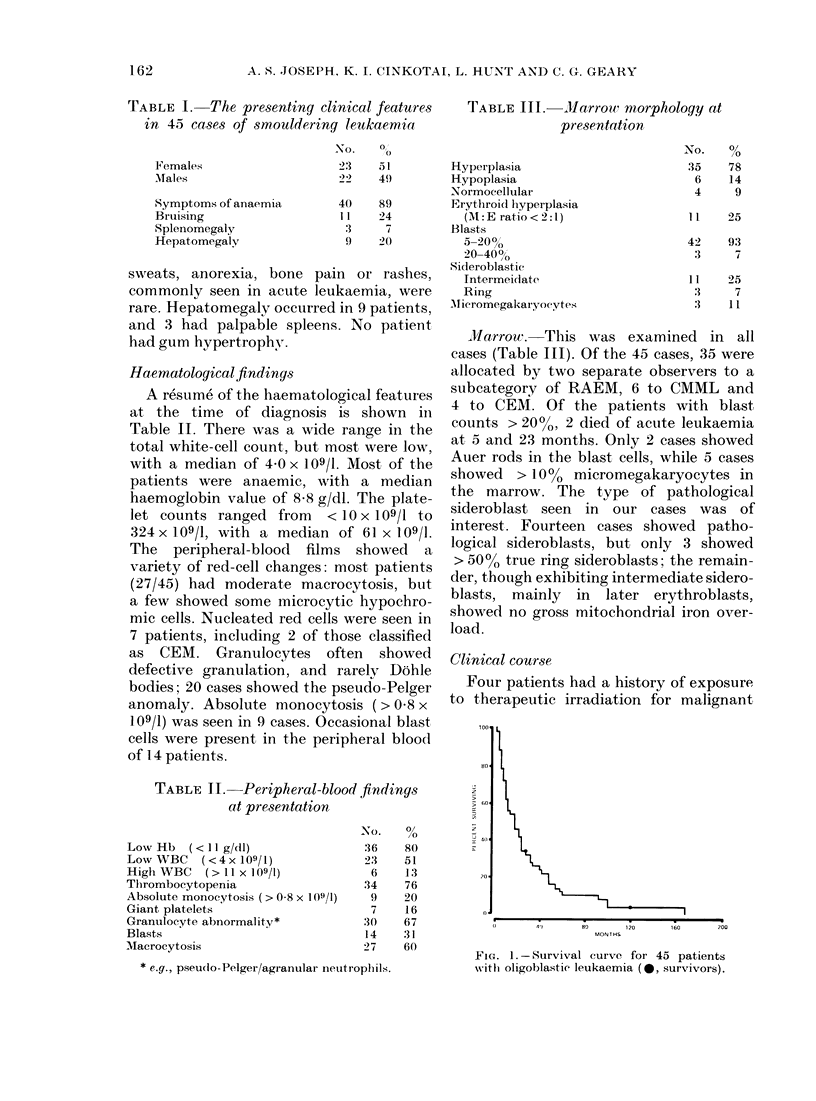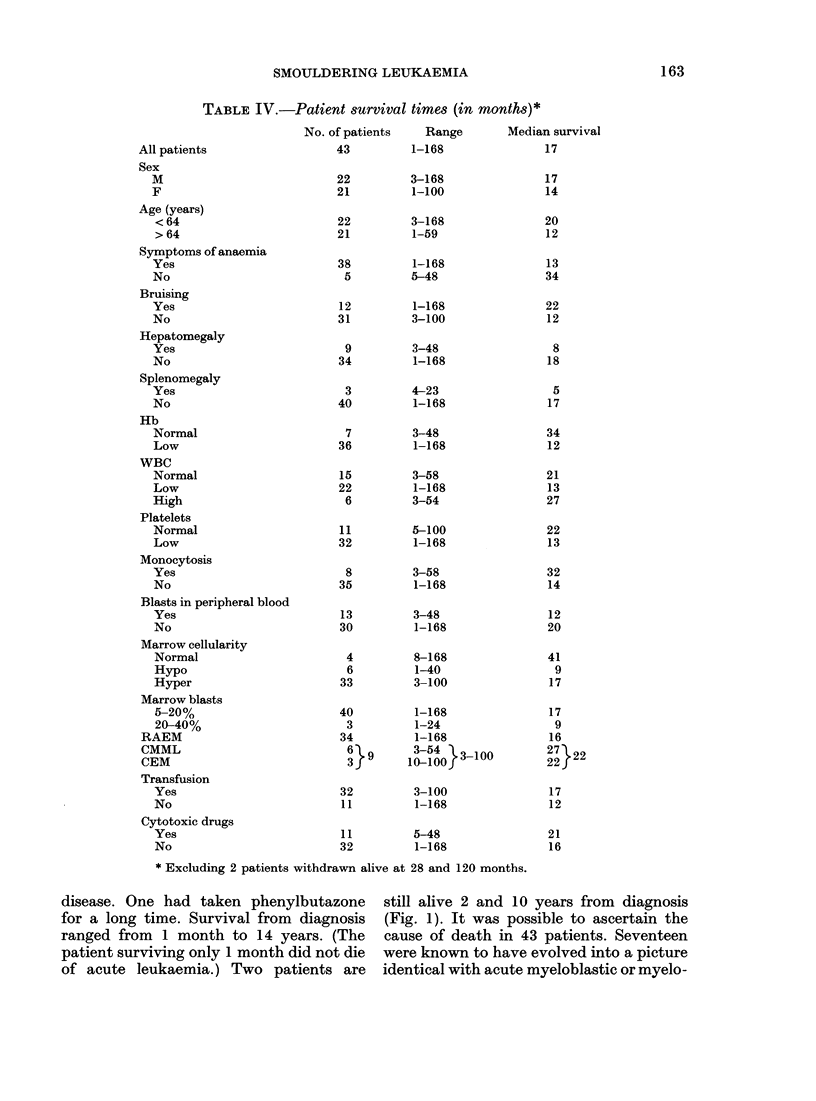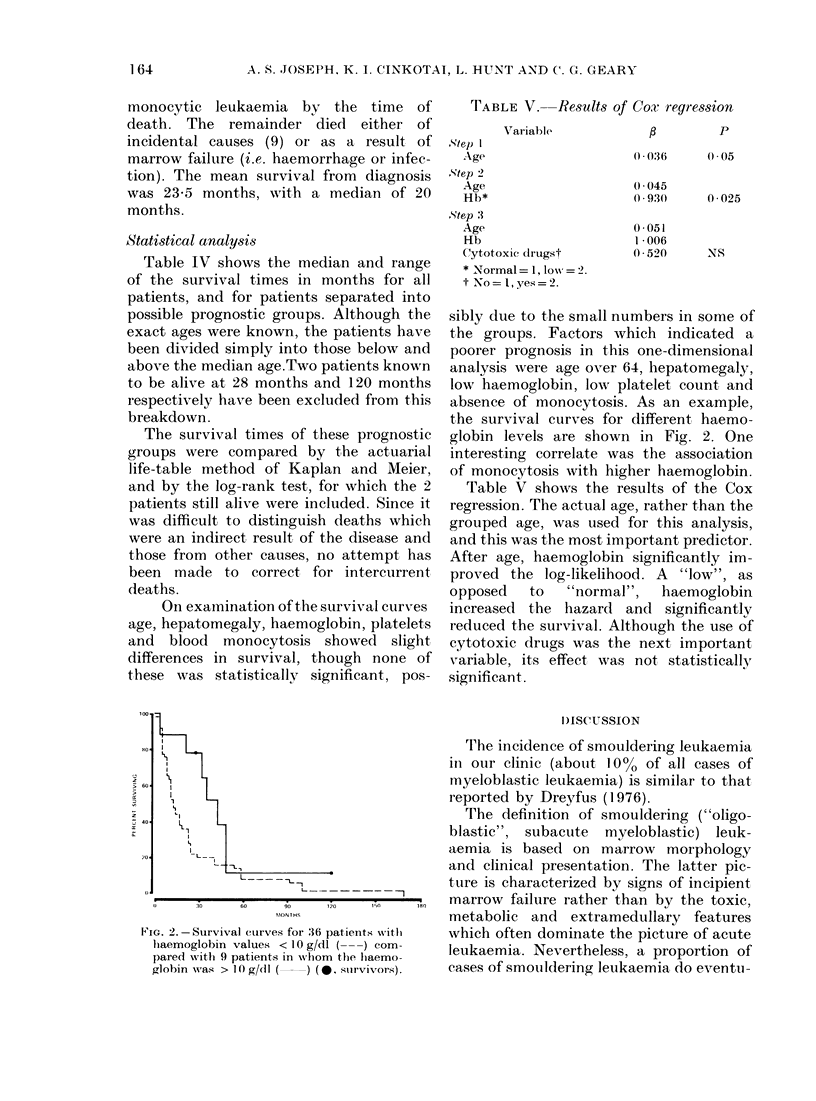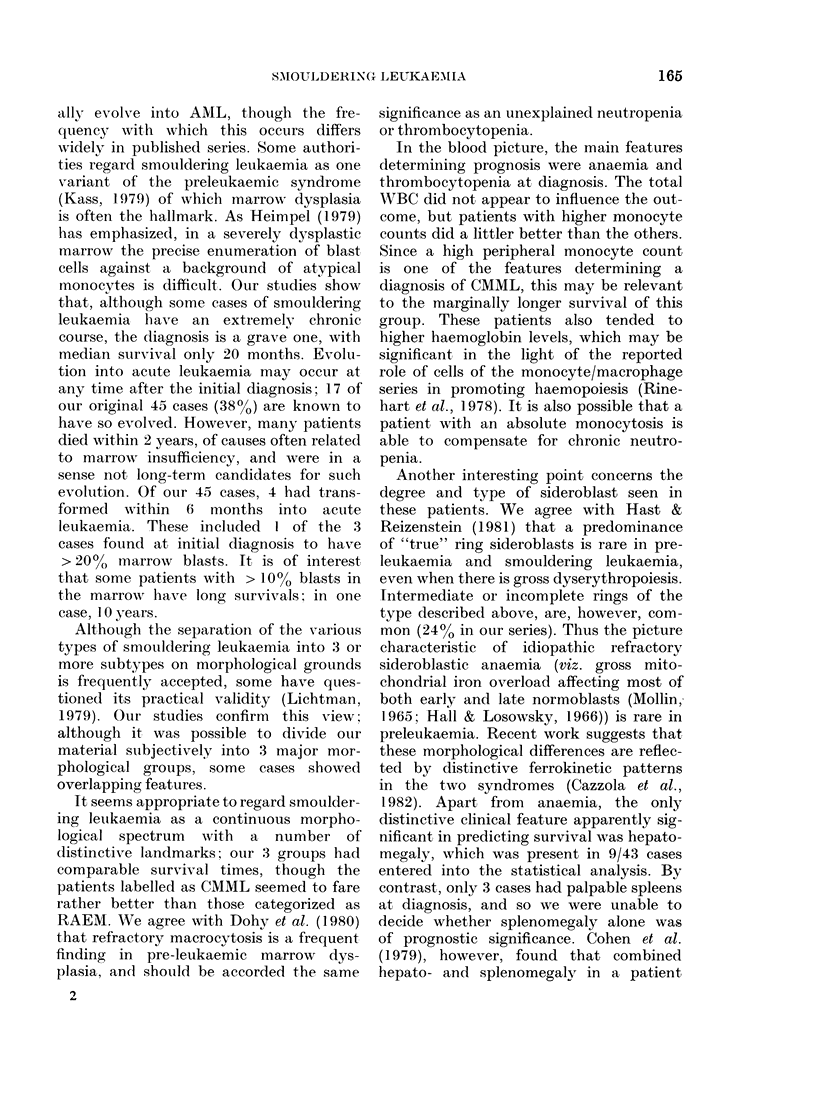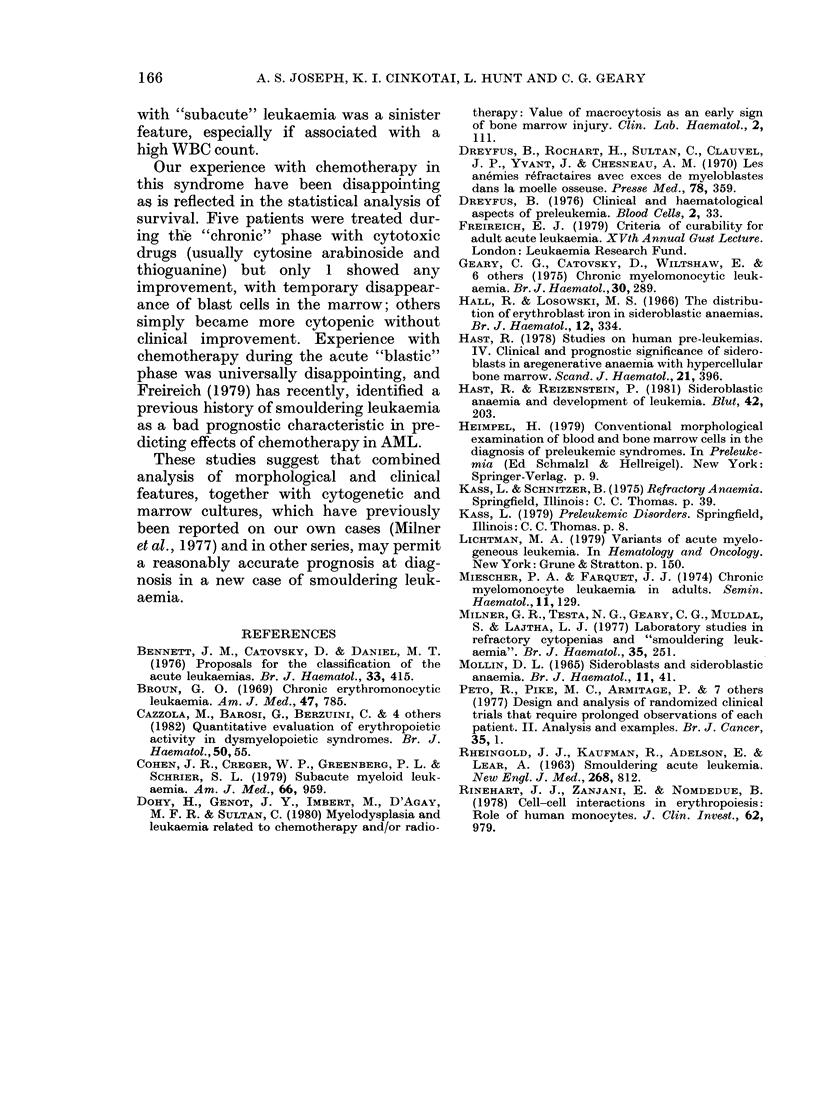# Natural history of smouldering leukaemia.

**DOI:** 10.1038/bjc.1982.179

**Published:** 1982-08

**Authors:** A. S. Joseph, K. I. Cinkotai, L. Hunt, C. G. Geary

## Abstract

The natural history of 45 cases of smouldering leukaemia has been studied. Males and females were equally represented, with a median age of 60.5. The median survival of the whole group was only 20 months, but rare cases lived 10 years or longer. 38% developed acute leukaemia; the remainder usually died of the results of marrow failure. Although it was possible to divide these marrow dysplasias morphologically into 3 major subgroups (refractory anaemia with excess of myeloblasts, chronic myelomonocytic leukaemia and chronic erythraemic myelosis), several displayed transitional features. Many showed refractory macrocytosis at diagnosis. The survival of the 3 groups was similar, though patients with high monocyte counts tended to present with less anaemia and fared rather better than the others. Statistical analysis suggests that increasing age, severe anaemia, thrombocytopenia and hepatomegaly are associated with a poor prognosis. Chemotherapy, when attempted, was usually unsuccessful.


*Br. J. Cancer* (1982) **46**, 160

### NATURAL HISTORY OF SMOULDERING LEUKAEMIA

A. S. JOSEPH, K. I. CINKOTAI, L. HUNT AND C. G. GEARY

*From the Department of Clinical Haematology and Faculty of Medicine Computational Group, University of Manchester, M13 9WL*

Received 7 December 1981 Accepted 6 April 1982

**Summary.—The natural history of 45 cases of smouldering leukaemia has been studied. Males and females were equally represented, with a median age of 60·5. The median survival of the whole group was only 20 months, but rare cases lived 10 years or longer. 38% developed acute leukaemia; the remainder usually died of the results of marrow failure. Although it was possible to divide these marrow dysplasias morphologically into 3 major subgroups (refractory anaemia with excess of myeloblasts, chronic myelomonocytic leukaemia and chronic erythraemic myelosis), several displayed transitional features. Many showed refractory macrocytosis at diagnosis. The survival of the 3 groups was similar, though patients with high monocyte counts tended to present with less anaemia and fared rather better than the others. Statistical analysis suggests that increasing age, severe anaemia, thrombocytopenia and hepatomegaly are associated with a poor prognosis. Chemotherapy, when attempted, was usually unsuccessful.**

THE TERM smouldering ("oligoblastic") leukaemia was introduced by Rheingold *et al.* (1963) to distinguish a group of patients, usually presenting with single or multiple cytopenias, whose marrows, while showing some of the maturation defects of acute myeloblastic or myelomonocytic leukaemia, differ from these in having a lower proportion of marrow blast cells. Clinically, the condition is more indolent than acute leukaemia, and particularly affects older individuals. A number of descriptive morphological terms have been applied to this syndrome, of which refractory anaemia with excess of myeloblasts (RAEM) (Dreyfus *et al.*, 1970), chronic myelomonocytic leukaemia (CMML) (Miescher & Farquet, 1974; Geary *et al.*, 1975), chronic erythraemic myelosis (CEM) (Kass & Schnitzer, 1975) and, less commonly, chronic erythromonocytic leukaemia (Broun, 1969) are among those used. However, it has been questioned whether subdivision into such categories is valid, and whether they have any clinical relevance.

In this study, 45 cases of smouldering leukaemia investigated in a single clinic have been followed for periods of up to 14 years, in an attempt to identify clinical and/or haematological features of possible prognostic significance. We also attempted to assess the validity of classifying our cases into 3 of the morphological types mentioned above.

### MATERIALS AND METHODS

We attempted to collect the records of all patients with a diagnosis of smouldering or "subacute" myeloblastic leukaemia seen in the University Department of Clinical Haematology at Manchester Royal Infirmary between 1971 and 1976. Since these titles are not yet included in the International Classification of Diseases, a few cases may have been included under other categories and thus overlooked. Eventually, 47 cases were identified, of which one was initially referred in 1962 and subsequently reclassified as a case of smouldering leukaemia. Two were discarded because of inadequate documentation. During this time, ~480 cases of myeloblastic leukaemia (AML) and its morphological variants were investigated and treated in the Department. An arbitrary level $\geqslant 5\%$ blasts in the marrow was chosen as identifying smouldering leukaemia. This figure is usually cited in the literature as discriminating leukaemic from dysmyelopoietic disorders, which may not necessarily be leukaemic. The upper limit was chosen as 40%, below which a diagnosis of smouldering leukaemia is tenable, in accordance with the FAB classification (Bennett *et al.*, 1976). In the blood, the following parameters were assessed: degree of anaemia, neutropenia, monocytosis and thrombocytopenia, and the presence of myeloblasts or nucleated red cells. In addition to the proportion of blast cells, the following morphological features were evaluated in the marrow: degree of monocytosis, number of megakaryocytes and their morphology, and the degree of dyserythropoiesis, with particular reference to the proportion of multinucleated erythroid precursors, and to the type and number of sideroblasts, as described by Mollin (1965) and Hast (1978). Marrow smears were stained with Prussian blue for the demonstration of non-haem iron in the erythroblasts. "Ringed" sideroblasts were defined as erythroblasts with large siderotic granules arranged in a perinuclear ring or collar, covering at least one-third of the circumference of the nucleus. "Intermediate" sideroblasts were defined as normoblasts with more than 6 non-haem iron granules, but diffusely scattered in the cytoplasm. Only patients with a predominance of "rings" (*i.e.* $>50\%$ of the total number of sideroblasts) were included in the ringed sideroblast group. The "intermediate" group consisted of the remaining patients, with mainly intermediate sideroblasts.

On the basis of these morphological features, in blood and marrow, the patients were allocated to one of the 3 main types of oligoblastic leukaemia: RAEM, CMML and CEM. In some cases, marrow cultures for CFU-C, CFU-E and cytogenetic analysis were undertaken. The prognostic value of these has already been reported (Milner *et al.*, 1977).

Clinically, information was sought about age, sex, length of history at diagnosis, evidence of exposure to potential marrow toxins, physical signs at diagnosis, subsequent clinical course, and survival. When patients died at home, or at another hospital, an attempt was made to ascertain the mode of death from information obtained from the patient's family doctor or consultant physician.

*Statistical methods*

Details for each patient were put on to coding forms and, after input to computer, were analysed in two ways:

(1) The effect of each variable (age, sex, WBC, etc.) was analysed individually by calculating Kaplan–Meier survival curves, which were then compared using the log-rank test (Peto *et al.*, 1977). For conuous variables, a convenient number of categories was first of all defined, and a log-rank $P$ value for trend calculated.

(2) The Cox regression method was used to detect prognostic variables. This multivariate analysis has the advantage that the merit of each variable at each stage is assessed, whilst correcting for the effects of any other variables in the analysis. If hazard function $\lambda(t)$ is the chance of dying in month $t$ in the patients alive at the beginning of the month, we assume

$$\lambda(t) = e^{a}\lambda_0(t)$$

where $\lambda_0(t)$ is a standard hazard function and $a = z_1\beta_1 + z_2\beta_2 + \ldots + z_p\beta_p$ where the $z$'s are a set of $p$ prognostic variables and the $\beta$'s are coefficients estimated by a maximum-likelihood iterative procedure. A stepwise procedure was adopted. First, the most important single predictor was found, and the next variable to be added to the analysis was the one which most improved the log-likelihood. More variables were added until none of them could significantly improve the log-likelihood.

### RESULTS

The age range of the patients was 44–77 years, with a median of 60·5. Males and females were almost equally represented. The presenting signs and symptoms are shown in Table I. The most frequently encountered symptoms were those attributable to chronic anaemia, but 24% also had a history of unusual bruising or bleeding. Recurrent infections were rare, but 2 patients had a history of oral ulceration. Toxic symptoms such as fever,

TABLE I.—*The presenting clinical features in 45 cases of smouldering leukaemia*

| | No. | % |
|---|---|---|
| Females | 23 | 51 |
| Males | 22 | 49 |
| | | |
| Symptoms of anaemia | 40 | 89 |
| Bruising | 11 | 24 |
| Splenomegaly | 3 | 7 |
| Hepatomegaly | 9 | 20 |

sweats, anorexia, bone pain or rashes, commonly seen in acute leukaemia, were rare. Hepatomegaly occurred in 9 patients, and 3 had palpable spleens. No patient had gum hypertrophy.

*Haematological findings*

A résumé of the haematological features at the time of diagnosis is shown in Table II. There was a wide range in the total white-cell count, but most were low, with a median of $4{\cdot}0 \times 10^9$/l. Most of the patients were anaemic, with a median haemoglobin value of 8·8 g/dl. The platelet counts ranged from $<10 \times 10^9$/l to $324 \times 10^9$/l, with a median of $61 \times 10^9$/l. The peripheral-blood films showed a variety of red-cell changes: most patients (27/45) had moderate macrocytosis, but a few showed some microcytic hypochromic cells. Nucleated red cells were seen in 7 patients, including 2 of those classified as CEM. Granulocytes often showed defective granulation, and rarely Döhle bodies; 20 cases showed the pseudo-Pelger anomaly. Absolute monocytosis ($>0{\cdot}8 \times 10^9$/l) was seen in 9 cases. Occasional blast cells were present in the peripheral blood of 14 patients.

TABLE II.—*Peripheral-blood findings at presentation*

| | No. | % |
|---|---|---|
| Low Hb ($<11$ g/dl) | 36 | 80 |
| Low WBC ($<4 \times 10^9$/l) | 23 | 51 |
| High WBC ($>11 \times 10^9$/l) | 6 | 13 |
| Thrombocytopenia | 34 | 76 |
| Absolute monocytosis ($>0{\cdot}8 \times 10^9$/l) | 9 | 20 |
| Giant platelets | 7 | 16 |
| Granulocyte abnormality* | 30 | 67 |
| Blasts | 14 | 31 |
| Macrocytosis | 27 | 60 |

* *e.g.*, pseudo-Pelger/agranular neutrophils.

TABLE III.—*Marrow morphology at presentation*

| | No. | % |
|---|---|---|
| Hyperplasia | 35 | 78 |
| Hypoplasia | 6 | 14 |
| Normocellular | 4 | 9 |
| Erythroid hyperplasia | | |
| (M:E ratio < 2:1) | 11 | 25 |
| Blasts | | |
| 5–20% | 42 | 93 |
| 20–40% | 3 | 7 |
| Sideroblastic | | |
| Intermeidate | 11 | 25 |
| Ring | 3 | 7 |
| Micromegakaryocytes | 3 | 11 |

*Marrow*.—This was examined in all cases (Table III). Of the 45 cases, 35 were allocated by two separate observers to a subcategory of RAEM, 6 to CMML and 4 to CEM. Of the patients with blast counts $>20\%$, 2 died of acute leukaemia at 5 and 23 months. Only 2 cases showed Auer rods in the blast cells, while 5 cases showed $>10\%$ micromegakaryocytes in the marrow. The type of pathological sideroblast seen in our cases was of interest. Fourteen cases showed pathological sideroblasts, but only 3 showed $>50\%$ true ring sideroblasts; the remainder, though exhibiting intermediate sideroblasts, mainly in later erythroblasts, showed no gross mitochondrial iron overload.

*Clinical course*

Four patients had a history of exposure to therapeutic irradiation for malignant

FIG. 1.—Survival curve for 45 patients with oligoblastic leukaemia (●, survivors).

TABLE IV.—*Patient survival times (in months)**

| | No. of patients | Range | Median survival |
|---|---|---|---|
| All patients | 43 | 1–168 | 17 |
| Sex | | | |
| M | 22 | 3–168 | 17 |
| F | 21 | 1–100 | 14 |
| Age (years) | | | |
| < 64 | 22 | 3–168 | 20 |
| > 64 | 21 | 1–59 | 12 |
| Symptoms of anaemia | | | |
| Yes | 38 | 1–168 | 13 |
| No | 5 | 5–48 | 34 |
| Bruising | | | |
| Yes | 12 | 1–168 | 22 |
| No | 31 | 3–100 | 12 |
| Hepatomegaly | | | |
| Yes | 9 | 3–48 | 8 |
| No | 34 | 1–168 | 18 |
| Splenomegaly | | | |
| Yes | 3 | 4–23 | 5 |
| No | 40 | 1–168 | 17 |
| Hb | | | |
| Normal | 7 | 3–48 | 34 |
| Low | 36 | 1–168 | 12 |
| WBC | | | |
| Normal | 15 | 3–58 | 21 |
| Low | 22 | 1–168 | 13 |
| High | 6 | 3–54 | 27 |
| Platelets | | | |
| Normal | 11 | 5–100 | 22 |
| Low | 32 | 1–168 | 13 |
| Monocytosis | | | |
| Yes | 8 | 3–58 | 32 |
| No | 35 | 1–168 | 14 |
| Blasts in peripheral blood | | | |
| Yes | 13 | 3–48 | 12 |
| No | 30 | 1–168 | 20 |
| Marrow cellularity | | | |
| Normal | 4 | 8–168 | 41 |
| Hypo | 6 | 1–40 | 9 |
| Hyper | 33 | 3–100 | 17 |
| Marrow blasts | | | |
| 5–20% | 40 | 1–168 | 17 |
| 20–40% | 3 | 1–24 | 9 |
| RAEM | 34 | 1–168 | 16 |
| CMML | 6 } 9 | 3–54 } 3–100 | 27 } 22 |
| CEM | 3 | 10–100 | 22 |
| Transfusion | | | |
| Yes | 32 | 3–100 | 17 |
| No | 11 | 1–168 | 12 |
| Cytotoxic drugs | | | |
| Yes | 11 | 5–48 | 21 |
| No | 32 | 1–168 | 16 |

* Excluding 2 patients withdrawn alive at 28 and 120 months.

disease. One had taken phenylbutazone for a long time. Survival from diagnosis ranged from 1 month to 14 years. (The patient surviving only 1 month did not die of acute leukaemia.) Two patients are still alive 2 and 10 years from diagnosis (Fig. 1). It was possible to ascertain the cause of death in 43 patients. Seventeen were known to have evolved into a picture identical with acute myeloblastic or myelomonocytic leukaemia by the time of death. The remainder died either of incidental causes (9) or as a result of marrow failure (*i.e.* haemorrhage or infection). The mean survival from diagnosis was 23·5 months, with a median of 20 months.

*Statistical analysis*

Table IV shows the median and range of the survival times in months for all patients, and for patients separated into possible prognostic groups. Although the exact ages were known, the patients have been divided simply into those below and above the median age.Two patients known to be alive at 28 months and 120 months respectively have been excluded from this breakdown.

The survival times of these prognostic groups were compared by the actuarial life-table method of Kaplan and Meier, and by the log-rank test, for which the 2 patients still alive were included. Since it was difficult to distinguish deaths which were an indirect result of the disease and those from other causes, no attempt has been made to correct for intercurrent deaths.

On examination of the survival curves age, hepatomegaly, haemoglobin, platelets and blood monocytosis showed slight differences in survival, though none of these was statistically significant, possibly due to the small numbers in some of the groups. Factors which indicated a poorer prognosis in this one-dimensional analysis were age over 64, hepatomegaly, low haemoglobin, low platelet count and absence of monocytosis. As an example, the survival curves for different haemoglobin levels are shown in Fig. 2. One interesting correlate was the association of monocytosis with higher haemoglobin.

TABLE V.—*Results of Cox regression*

| Variable | β | P |
|---|---|---|
| *Step* 1 | | |
| Age | 0·036 | 0·05 |
| *Step* 2 | | |
| Age | 0·045 | |
| Hb* | 0·930 | 0·025 |
| *Step* 3 | | |
| Age | 0·051 | |
| Hb | 1·006 | |
| Cytotoxic drugs† | 0·520 | NS |

* Normal = 1, low = 2.

† No = 1, yes = 2.

Table V shows the results of the Cox regression. The actual age, rather than the grouped age, was used for this analysis, and this was the most important predictor. After age, haemoglobin significantly improved the log-likelihood. A "low", as opposed to "normal", haemoglobin increased the hazard and significantly reduced the survival. Although the use of cytotoxic drugs was the next important variable, its effect was not statistically significant.

FIG. 2.—Survival curves for 36 patients with haemoglobin values < 10 g/dl (– – –) compared with 9 patients in whom the haemoglobin was > 10 g/dl (——) (●, survivors).

DISCUSSION

The incidence of smouldering leukaemia in our clinic (about 10% of all cases of myeloblastic leukaemia) is similar to that reported by Dreyfus (1976).

The definition of smouldering ("oligoblastic", subacute myeloblastic) leukaemia is based on marrow morphology and clinical presentation. The latter picture is characterized by signs of incipient marrow failure rather than by the toxic, metabolic and extramedullary features which often dominate the picture of acute leukaemia. Nevertheless, a proportion of cases of smouldering leukaemia do eventually evolve into AML, though the frequency with which this occurs differs widely in published series. Some authorities regard smouldering leukaemia as one variant of the preleukaemic syndrome (Kass, 1979) of which marrow dysplasia is often the hallmark. As Heimpel (1979) has emphasized, in a severely dysplastic marrow the precise enumeration of blast cells against a background of atypical monocytes is difficult. Our studies show that, although some cases of smouldering leukaemia have an extremely chronic course, the diagnosis is a grave one, with median survival only 20 months. Evolution into acute leukaemia may occur at any time after the initial diagnosis; 17 of our original 45 cases (38%) are known to have so evolved. However, many patients died within 2 years, of causes often related to marrow insufficiency, and were in a sense not long-term candidates for such evolution. Of our 45 cases, 4 had transformed within 6 months into acute leukaemia. These included 1 of the 3 cases found at initial diagnosis to have $>20\%$ marrow blasts. It is of interest that some patients with $>10\%$ blasts in the marrow have long survivals; in one case, 10 years.

Although the separation of the various types of smouldering leukaemia into 3 or more subtypes on morphological grounds is frequently accepted, some have questioned its practical validity (Lichtman, 1979). Our studies confirm this view; although it was possible to divide our material subjectively into 3 major morphological groups, some cases showed overlapping features.

It seems appropriate to regard smouldering leukaemia as a continuous morphological spectrum with a number of distinctive landmarks; our 3 groups had comparable survival times, though the patients labelled as CMML seemed to fare rather better than those categorized as RAEM. We agree with Dohy *et al.* (1980) that refractory macrocytosis is a frequent finding in pre-leukaemic marrow dysplasia, and should be accorded the same significance as an unexplained neutropenia or thrombocytopenia.

In the blood picture, the main features determining prognosis were anaemia and thrombocytopenia at diagnosis. The total WBC did not appear to influence the outcome, but patients with higher monocyte counts did a littler better than the others. Since a high peripheral monocyte count is one of the features determining a diagnosis of CMML, this may be relevant to the marginally longer survival of this group. These patients also tended to higher haemoglobin levels, which may be significant in the light of the reported role of cells of the monocyte/macrophage series in promoting haemopoiesis (Rinehart *et al.*, 1978). It is also possible that a patient with an absolute monocytosis is able to compensate for chronic neutropenia.

Another interesting point concerns the degree and type of sideroblast seen in these patients. We agree with Hast & Reizenstein (1981) that a predominance of "true" ring sideroblasts is rare in preleukaemia and smouldering leukaemia, even when there is gross dyserythropoiesis. Intermediate or incomplete rings of the type described above, are, however, common (24% in our series). Thus the picture characteristic of idiopathic refractory sideroblastic anaemia (*viz.* gross mitochondrial iron overload affecting most of both early and late normoblasts (Mollin, 1965; Hall & Losowsky, 1966)) is rare in preleukaemia. Recent work suggests that these morphological differences are reflected by distinctive ferrokinetic patterns in the two syndromes (Cazzola *et al.*, 1982). Apart from anaemia, the only distinctive clinical feature apparently significant in predicting survival was hepatomegaly, which was present in 9/43 cases entered into the statistical analysis. By contrast, only 3 cases had palpable spleens at diagnosis, and so we were unable to decide whether splenomegaly alone was of prognostic significance. Cohen *et al.* (1979), however, found that combined hepato- and splenomegaly in a patient with "subacute" leukaemia was a sinister feature, especially if associated with a high WBC count.

Our experience with chemotherapy in this syndrome have been disappointing as is reflected in the statistical analysis of survival. Five patients were treated during the "chronic" phase with cytotoxic drugs (usually cytosine arabinoside and thioguanine) but only 1 showed any improvement, with temporary disappearance of blast cells in the marrow; others simply became more cytopenic without clinical improvement. Experience with chemotherapy during the acute "blastic" phase was universally disappointing, and Freireich (1979) has recently, identified a previous history of smouldering leukaemia as a bad prognostic characteristic in predicting effects of chemotherapy in AML.

These studies suggest that combined analysis of morphological and clinical features, together with cytogenetic and marrow cultures, which have previously been reported on our own cases (Milner *et al.*, 1977) and in other series, may permit a reasonably accurate prognosis at diagnosis in a new case of smouldering leukaemia.

### REFERENCES

Bennett, J. M., Catovsky, D. & Daniel, M. T. (1976) Proposals for the classification of the acute leukaemias. *Br. J. Haematol.*, **33**, 415.

Broun, G. O. (1969) Chronic erythromonocytic leukaemia. *Am. J. Med.*, **47**, 785.

Cazzola, M., Barosi, G., Berzuini, C. & 4 others (1982) Quantitative evaluation of erythropoietic activity in dysmyelopoietic syndromes. *Br. J. Haematol.*, **50**, 55.

Cohen, J. R., Creger, W. P., Greenberg, P. L. & Schrier, S. L. (1979) Subacute myeloid leukaemia. *Am. J. Med.*, **66**, 959.

Dohy, H., Genot, J. Y., Imbert, M., D'Agay, M. F. R. & Sultan, C. (1980) Myelodysplasia and leukaemia related to chemotherapy and/or radiotherapy: Value of macrocytosis as an early sign of bone marrow injury. *Clin. Lab. Haematol.*, **2**, 111.

Dreyfus, B., Rochart, H., Sultan, C., Clauvel, J. P., Yvant, J. & Chesneau, A. M. (1970) Les anémies réfractaires avec exces de myeloblastes dans la moelle osseuse. *Presse Med.*, **78**, 359.

Dreyfus, B. (1976) Clinical and haematological aspects of preleukemia. *Blood Cells*, **2**, 33.

Freireich, E. J. (1979) Criteria of curability for adult acute leukaemia. *XVth Annual Gust Lecture.* London: Leukaemia Research Fund.

Geary, C. G., Catovsky, D., Wiltshaw, E. & 6 others (1975) Chronic myelomonocytic leukaemia. *Br. J. Haematol.*, **30**, 289.

Hall, R. & Losowski, M. S. (1966) The distribution of erythroblast iron in sideroblastic anaemias. *Br. J. Haematol.*, **12**, 334.

Hast, R. (1978) Studies on human pre-leukemias. IV. Clinical and prognostic significance of sideroblasts in aregenerative anaemia with hypercellular bone marrow. *Scand. J. Haematol.*, **21**, 396.

Hast, R. & Reizenstein, P. (1981) Sideroblastic anaemia and development of leukemia. *Blut*, **42**, 203.

Heimpel, H. (1979) Conventional morphological examination of blood and bone marrow cells in the diagnosis of preleukemic syndromes. In *Preleukemia* (Ed Schmalzl & Hellreigel). New York: Springer-Verlag. p. 9.

Kass, L. & Schnitzer, B. (1975) *Refractory Anaemia.* Springfield, Illinois: C. C. Thomas. p. 39.

Kass, L. (1979) *Preleukemic Disorders.* Springfield, Illinois: C. C. Thomas. p. 8.

Lichtman, M. A. (1979) Variants of acute myelogeneous leukemia. In *Hematology and Oncology.* New York: Grune & Stratton. p. 150.

Miescher, P. A. & Farquet, J. J. (1974) Chronic myelomonocyte leukaemia in adults. *Semin. Haematol.*, **11**, 129.

Milner, G. R., Testa, N. G., Geary, C. G., Muldal, S. & Lajtha, L. J. (1977) Laboratory studies in refractory cytopenias and "smouldering leukaemia". *Br. J. Haematol.*, **35**, 251.

Mollin, D. L. (1965) Sideroblasts and sideroblastic anaemia. *Br. J. Haematol.*, **11**, 41.

Peto, R., Pike, M. C., Armitage, P. & 7 others (1977) Design and analysis of randomized clinical trials that require prolonged observations of each patient. II. Analysis and examples. *Br. J. Cancer*, **35**, 1.

Rheingold, J. J., Kaufman, R., Adelson, E. & Lear, A. (1963) Smouldering acute leukemia. *New Engl. J. Med.*, **268**, 812.

Rinehart, J. J., Zanjani, E. & Nomdedue, B. (1978) Cell–cell interactions in erythropoiesis: Role of human monocytes. *J. Clin. Invest.*, **62**, 979.